# Compressive and Energy Absorption Properties of Pyramidal Lattice Structures by Various Preparation Methods

**DOI:** 10.3390/ma14216484

**Published:** 2021-10-28

**Authors:** Hairi Zhang, Xingfu Wang, Zimu Shi, Jintao Xue, Fusheng Han

**Affiliations:** 1 Key Laboratory of Materials Physics, Institute of Solid State Physics, Hefei Institutes of Physical Science, Chinese Academy of Sciences, Hefei 230031, China; wangxingfu@issp.ac.cn (X.W.); shizimu@issp.ac.cn (Z.S.); jintxue@163.com (J.X.); 2 Science Island Branch, Graduate School of USTC, Hefei 230026, China

**Keywords:** lattice structures, energy absorption, finite element analysis, compression behavior, additive manufacturing, investment casting

## Abstract

Metallic three-dimensional lattice structures exhibit many favorable mechanical properties including high specific strength, high mechanical efficiency and superior energy absorption capability, being prospective in a variety of engineering fields such as light aerospace and transportation structures as well as impact protection apparatus. In order to further compare the mechanical properties and better understand the energy absorption characteristics of metal lattice structures, enhanced pyramidal lattice structures of three strut materials was prepared by 3D printing combined with investment casting and direct metal additive manufacturing. The compressive behavior and energy absorption property are theoretically analyzed by finite element simulation and verified by experiments. It is shown that the manufacturing method of 3D printing combined with investment casting eliminates stress fluctuations in plateau stages. The relatively ideal structure is given by examination of stress–strain behavior of lattice structures with varied parameters. Moreover, the theoretical equation of compressive strength is established that can predicts equivalent modulus and absorbed energy of lattice structures.

## 1. Introduction

Cellular materials have become an important structural and functional material due to their low density, high specific strength and porous structure, etc. [1,2]. Conventional cellular materials are mainly foams, sponges and honeycombs, which are usually used for functional purposes such as sound barrier, vibration isolating apparatus and impact protection devices [3,4,5]. For the metallic foams and sponges, the pore structures including pore shape, size, number and distribution are intrinsically random, or in other words, they are almost not controllable, while for the honeycombs, the pore distribution and mechanical properties are strongly anisotropic. These characteristics, to a certain degree, limit the applications of conventional metallic cellular materials. Recently developed three-dimensional lattice structures would be one of the most ideal solutions to these problems. They have exactly designed cell structures, almost unlimited matrix materials, optimized properties, and can be manufactured by many industrially scaled technologies. This makes them prospective candidates in many engineering fields, for example, in aviation, aerospace, and automotive industries where high and controllable mechanical properties are needed [6,7,8].

One of the most important properties of cellular materials is energy absorption capacity characterized by absorbed or dissipated mechanical energy during compression. It has been verified that most lattice structures, such as pyramidal, Kagome, diamond and Re-entrant, etc., have very excellent energy absorption properties [9,10,11,12,13] that can be designed by changing the geometric parameters, such as diameter-to-length ratio [14] or unit cell size [15]. Moreover, the sandwich panels with lattice structures core that can be designed are also proven to have excellent energy absorption properties [16]. In addition to geometric parameters, the energy absorption properties can also be tailored by complex structures such as gradient, metal foam filled, multi-layered and different unit cell composed structures. They have been demonstrated to be more effective than single lattice structures in energy absorption [17,18,19,20]. Another method to enhance the mechanical properties or energy absorption capacity is to modify the structure of node connecting the struts, where the stress concentration usually arises when subjected to compression or impact load. This method is relatively simple but very effective in increasing the energy absorption capacity of metallic lattice structures [21,22].

There have been several technologies to manufacture metallic lattice structures so far including stamping forming [23], extrusion combining wire cutting [24], expanded sheet folding [25], and investment casting [26]. Aside from casting, these technologies have to use bonding or welding techniques to assemble the struts to form lattice structures. During processing, the junctions are usually defect sensitive, such as to gas bubbles and micro-cracks, causing the overall mechanical properties of lattice structures to be weakened. For the investment casting technology, the cell configuration cannot be too complex because of the limitation of technology itself. Thanks to recently developed metallic additive manufacturing technologies, it makes the fabrication of even more complex metallic lattice structures possible without need of traditional bonding techniques. However, there are also some limitations in additive manufacturing technologies because selective laser sintering (SLS) or selective laser melting (SLM) has to be used for bonding [27,28]. This leads to only a small part of metals suitable for these technologies. To overcome the limitations of additive manufacturing and investment casting, a new technology has been developed in recent years that combines 3-D printing with investment casting. In this technology, a low melting point resin-based lattice structure is firstly prepared by 3-D printing, and then by using it as the pattern, a ceramic shell mold is produced. Finally, a molten metal is infiltrated into the cavity of mold under compressed air, and after the metal solidifies, the shell is removed by water rinsing, leaving a metal lattice structure. This method has almost no limitation in choosing matrix metals to produce lattice structures in addition to the ability to produce any complex configuration [29,30].

Currently, there are few studies on the differences in mechanical properties and energy absorption caused by different preparation methods of lattice structures. In this study, the pyramidal lattice structures in three strut materials are fabricated through two preparation methods. The influence of preparation methods and design parameters on their compression response and energy absorption are systematically studied through finite element analysis and compression test. Theoretical prediction formulas of compressive strength, equivalent modulus and energy absorption are proposed to provide theoretical guidance for the design of lattice structures.

## 2. Materials and Methods

### 2.1. Preparation of Lattice Structure Samples

Two technologies, direct 3D printing and 3D printing combined with investment casting, were used to prepare the lattice structure samples. In the former, a BLT-A300 printer (BLT Company, Xi’an, China) and selective laser melting (SLM) method were used. The laser power is 500 W, the scanning speed is 1500 mm/s, and the layer thickness is 50 μm. The hatch spacing is 0.10–0.19 mm. The material used in the printing is additive manufacturing (AM) AlSi10Mg powder with the mean particle size of 15–53 μm. In the latter, an as-cast AlSi10Mg alloy and a 7005 aluminum alloy were used and the following processes were experienced:

(1) A pattern of pyramidal lattice structure was firstly fabricated by a printer using photosensitive resin through stereo-lithography (SLA) technology.

(2) A plaster mold was fabricated by filling the plaster slurry in the photosensitive resin pattern fixed in a stainless steel container. After the plaster slurry was dried and hardened, it was heated to 500 °C and kept for 3 h to remove the photosensitive resin and thereby form the cavity of lattice structures.

(3) Pouring the liquid aluminum alloy into the container, quickly sealing it and then inputting compressed air to make the molten metal infiltrate into the cavity of mold. After the molten aluminum alloy solidified, the plaster mold was collapsed by a high-pressure water jet, leaving an aluminum alloy lattice structure.

**Figure 1:**
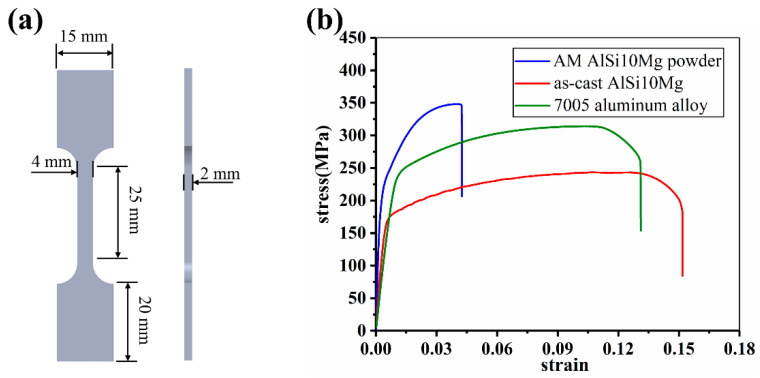
The tensile test of different aluminum alloys, (**a**) is the geometric dimensions of tensile sample; (**b**) is the stress–strain curves.

To know the mechanical properties of different aluminum alloys used in the present study, the tensile experiments were carried out. The geometric dimensions of samples used for the tensile test are shown in Figure 1a. The tensile rate is set to 2 mm/min, and a laser extensometer is used to characterize the strain. Each type of sample is tested three times, and the arithmetic mean value is taken as the representative value. The stress–strain curves of related alloys are shown in Figure 1b. Among the three alloys, the AM AlSi10Mg alloy has the highest strength and stiffness, but the lowest ductility. On the contrary, the as-cast AlSi10Mg alloy shows the highest ductility but the lowest strength. The 7005 aluminum alloy exhibits moderate strength and ductility, just between the other two.

### 2.2. Enhanced Pyramidal Lattice Structure

**Figure 2:**
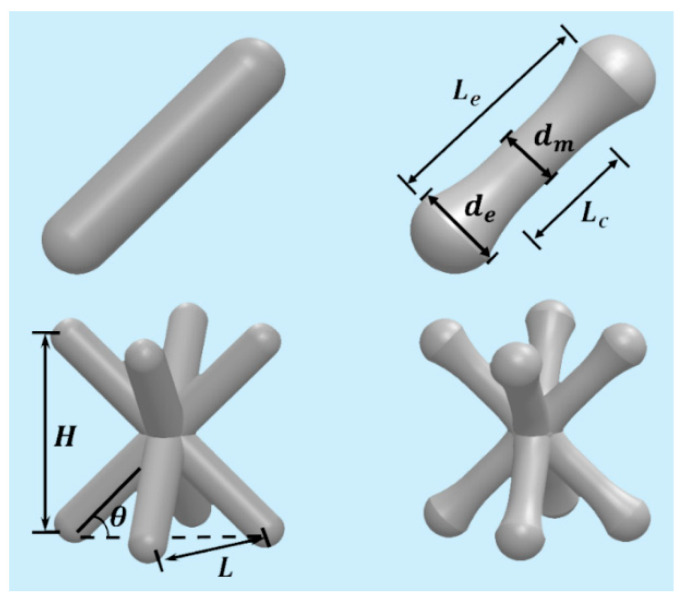
Characteristics of pyramidal lattice structures in the present study: the left is usual and the right is enhanced.

The mechanical properties of the lattice structure can be improved through node-enhancement [21,22]. In this study, the effect of preparation method and strut materials are the focus of attention. An enhanced pyramidal (EP) lattice structure and a usual one are shown in Figure 2. It is clear that the enhancement is realized simply by gradually increasing the diameter of struts toward the node.

The related geometric parameters are defined as follows: *d_e_* and *d_m_* represent the end and middle diameter of strut, respectively; *L*, *H* and *θ* represent the width of bottom plane, the height of unit cell and the included angle of strut with the bottom plane, respectively; *L_e_* and *L_c_* are the effective length and equal-diameter length of strut, respectively. To study the influence of geometric parameters, the value of *d_e_* varies from 1.4 mm to 1.8 mm with an interval of 0.1 mm, where 1.4 mm is also the diameter of usual pyramidal structure used for comparison. When *d_e_* is increased, *d_m_* is decreased to keep the relative density of lattice structure constant. The *θ* value varies from 35° to 55° with an interval of 10°.

**Figure 3:**
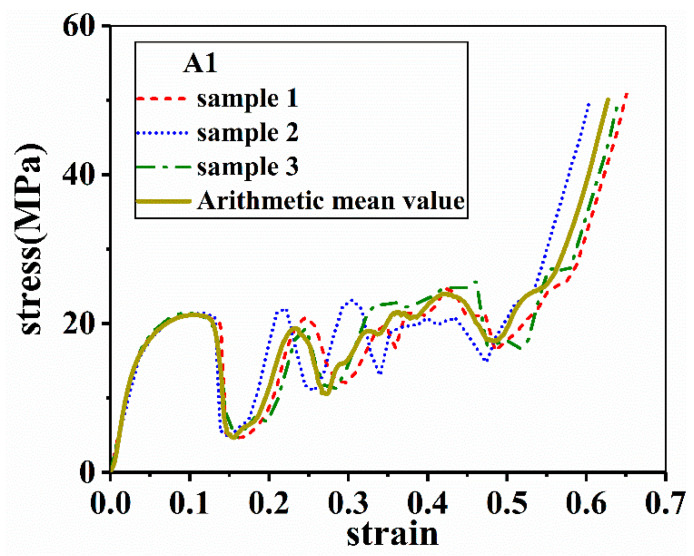
Arithmetic mean value of three experimental data.

The lattice structures are divided into four groups, as listed in Table 1. In order to control variables, all the samples have the same *L_e_* and *L_c_* that are 4.36 mm and 1.45 mm, respectively. In group A, three lattice structures are included with varied aluminum alloys as the matrix and a constant inclination angle of 35°. In group B and C, two inclination angles, i.e., 45° and 55°, are examined. In group D, only *d_e_* and corresponding *d_m_* are changed while the other parameters are kept constant. $\rho^{*}$ is the relative density of a sample. As shown in Figure 3, A1 is chosen as an example, at least three samples are tested for each structure, and the arithmetic mean value is taken as the representative value.

### 2.3. Finite Element Analysis

Finite element analysis (FEA) is conducted to simulate the mechanical response of lattice structures in compression. A three-dimensional homogeneous solid tetrahedron element with the element type of C3D10M is used to establish the model. Free meshing technique is adopted to solve the problem of complex geometries. The Poisson’s ratio of strut materials is set to 0.3, and the density is 2.7 g/cm^3^. The values of Young’s modulus and the plastic constitutive model of all strut materials are obtained from the tensile tests, the curves are shown in Figure 1b. The relevant plastic parameters are listed in Table 2. The Young’s modulus of three strut materials in the table from left to right are 79 GPa, 71 GPa and 67 GPa respectively. The number of elements of each model is about 130,000. The computational time is about 12 h. There are two rigid plates placed on the upper and lower sides of the model, respectively, and the element type is S4R. In order to completely simulate the collapse process of lattice structures before densification and take into account the calculation time, the simulation process end shortly after the densification stage begins. When the inclination angle is 35°, 45° and 55°, the compression strain of upper plate is set to 0.7, 0.8 and 0.9 by analyzing experimental data and theoretical calculations. The free degree of bottom plate is set to 0. General contact condition is adopted to solve the convergence problems. In order to simplify the calculation, only a half of the model is considered in the numerical analysis and symmetrical boundary condition is applied. The size of the element is 0.25 mm through the convergence analysis.

### 2.4. Compression Tests

**Figure 4:**
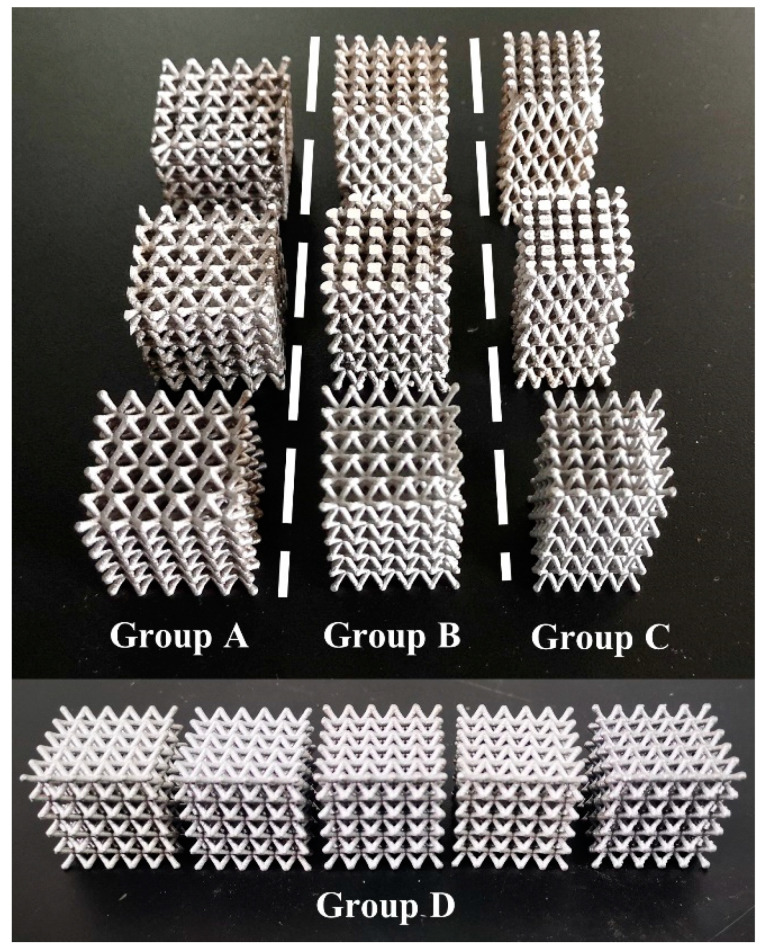
Four group samples, A, B, C and D, of node-enhanced pyramidal lattice structures.

Uniaxial compression tests were carried out in a material testing system (Instron 3369, Instron Corporation, Canton, OH, USA) with a displacement rate of 2 mm/min. The samples contained 5 × 5 × 5 unit cells, as shown in Figure 4, to reduce the size effect. Before the test, the surfaces of samples were slightly polished to guarantee good contact between the samples and the indenter. The deformation process of samples was recorded by a high-resolution digital camera.

## 3. Results and Discussion

### 3.1. Compression Behaviors of Samples

**Figure 5:**
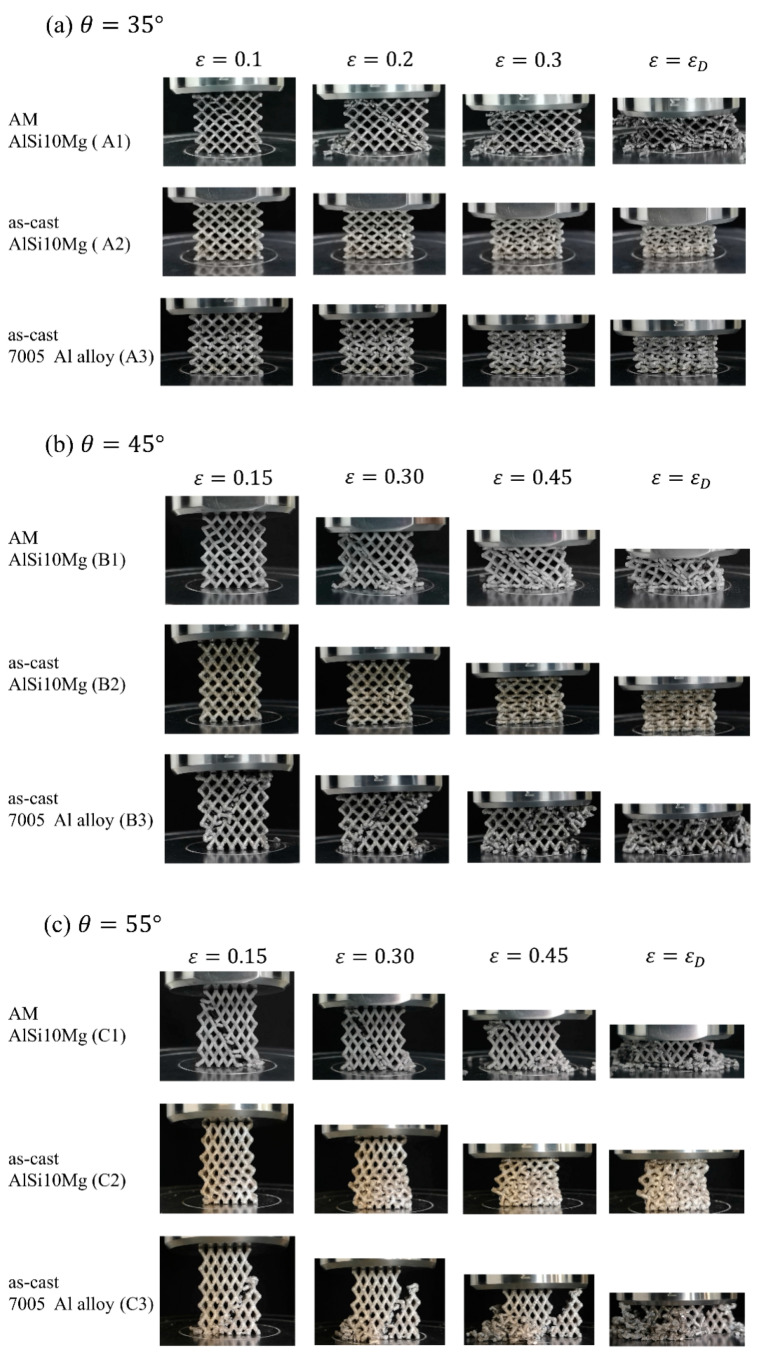
Deformation process of EP lattice structures in Group A, B and C; (**a**–**c**) represent the compression when the included angle is 35°, 45° and 55°, respectively.

**Figure 6:**
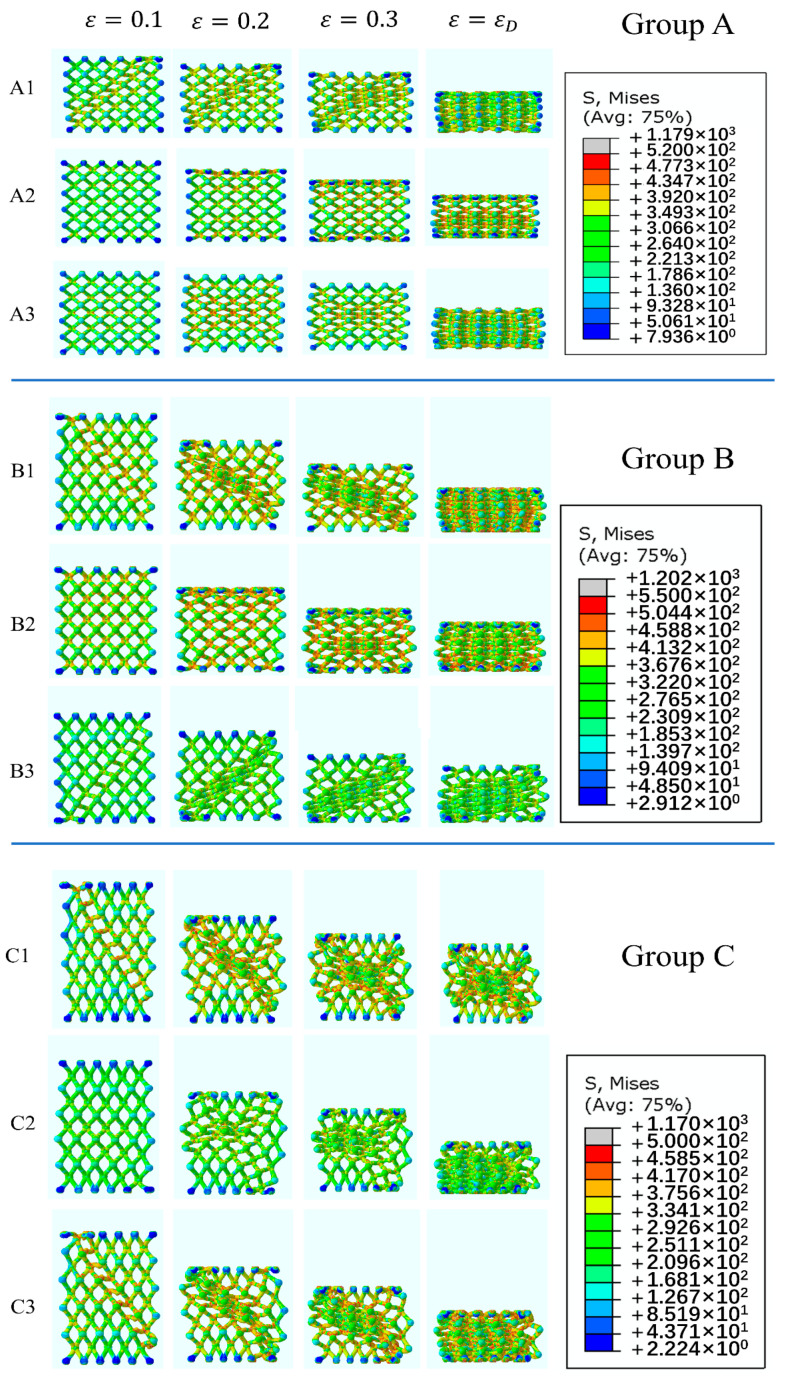
Mises stress distribution diagrams of EP lattice structures in Group A, B and C.

**Figure 7:**
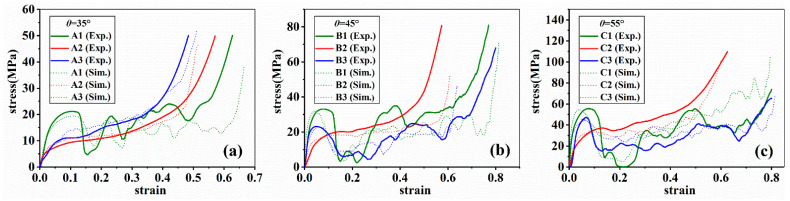
The stress–strain curves of samples; (**a**–**c**) represent Group A, B, and C respectively.

Figure 5 gives the deformation processes of samples in Group A, B and C, in which three different included angles and matrix material states were examined. Figure 6 and Figure 7 present the results of FEA and the corresponding stress–strain curves, respectively.

At the smallest included angle, 35°, the samples A2 and A3 show a similar deformation mode. The deformation started from the struts in the middle layer, and then the struts were bent and folded layer by layer with less lateral extension as the compression deformation proceeded until they were all pressed together. The sample A1, however, shows a very different deformation mode. There appeared a diagonal shearing band in the structure at the beginning of deformation. As the compression continued, the struts in the band were broken while those in other areas seemed to keep unchanged. Throughout the whole compression process, the structure was deformed and densified in the form of shearing along the band, leading to obvious lateral extension, as shown in Figure 5a.

When the included angle was increased to 45°, the samples B1 and B2 show a similar deformation behavior to A1 and A2, respectively, but the sample B3 shown a quite different deformation mode from A3. The bending started from the struts near the two planes up and down, and then gradually extended toward the middle area. At the same time, cracks were produced and propagated, causing the lattice structure to be collapsed, as shown in Figure 5b.

At the largest included angle, 55°, there was no obvious change in the deformation modes of C1 and C2, compared with that of B1 and B2. However, the deformation and failure characteristics of C3 were completely changed. Cracks were formed at the beginning of compression, and subsequently the structure was completely fractured when the strain was only 0.3, exhibiting a brittle nature, as shown in Figure 5c.

The simulated deformation evolutions shown in Figure 6 are approximately coincident with the experiment results shown in Figure 4. Stress concentration mainly occurs near the nodes. It is seen that stress concentration is the predominant reason for localized deformation and fracture. On the whole, the lattice structures based on as-cast AlSi10Mg alloy exhibit the most uniform stress distribution among the three alloys.

**Figure 8:**
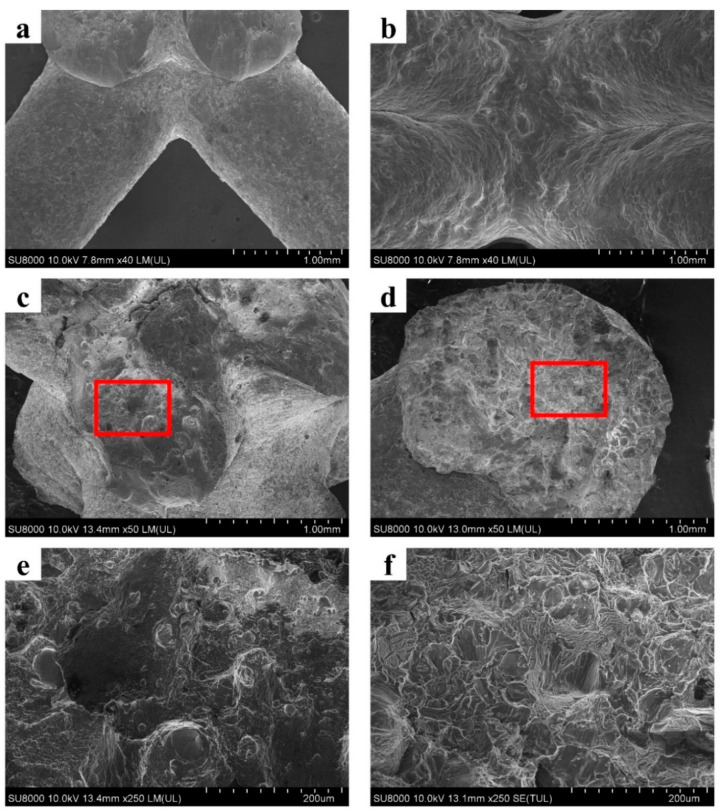
The strut surface morphology of (**a**) AM AlSi10Mg and (**b**) as-cast 7005 Al alloy lattice structures; (**c**,**d**) are the morphology of fracture surface of AM AlSi10Mg and as-cast 7005 Al alloy lattice structures; (**e**,**f**) magnified microstructures in the red frames of (**c**,**d**).

It could be noticed from Figure 7 that, in all situations, only as-cast AlSi10Mg alloy shows smooth stress strain curves. This is consistent with the deformation mode of as-cast AlSi10Mg alloy and stress distribution in it. As shown in Figure 5 and Figure 6, in the all included angles examined, the compression deformation of samples was characterized by folding layer by layer and no fracture or collapsing was seen in the struts. In addition, the stress distribution of as-cast AlSi10Mg alloy sample was uniform during the whole deformation process relative to other samples. Obviously, these differences have arisen because the as-cast AlSi10Mg alloy has the best ductility among the three alloys, as shown by Figure 1. These results suggest that the mechanical properties of strut material could be the decisive factor for the response behavior of related lattice structures while the other structural parameters could be less effective. This deduction can be further verified by the failure mechanisms shown in Figure 8. The surface of AM AlSi10Mg lattice structures is relatively flat, and occasionally some partially melted particles are attached. The surface of as-cast has many rippled patterns that cause certain undulations, which are caused by the residual photosensitive resin in the cavity. This undulation is small for the diameter of struts, therefore it does not significantly affect the mechanical properties [31]. It is seen that there are many dimples in the fracture surface of AM AlSi10Mg lattice structures, while there are a large number of cleavage surfaces appearing in the as-cast 7005 Al alloy lattice structures. Obviously, the former exhibits ductile while the latter shows brittle fracture, being consistent with the intrinsic mechanical properties of their strut materials.

Deshpande et al. [26] divided the deformation modes of lattice structures into two types, i.e., bending-dominated and stretching-dominated deformation in terms of load-bearing characteristics of struts. According to this definition, the deformation of sample A1 should belong to stretching-dominated while that of A2 and A3 should be bending-dominated mode. This conclusion can be easily understood if Figure 5 and Figure 6 are seen. At the beginning of compression, the stress of A1 rose rapidly until reaching a maximum value and then it sharply dropped. Accompanied with the changes of stress, a diagonal shearing band appeared in the lattice structure, which is a typical feature of tensile deformation. By contrast, there was no shearing band occurring in the structure, but the struts were folded layer by layer, typical for bending deformation.

**Figure 9:**
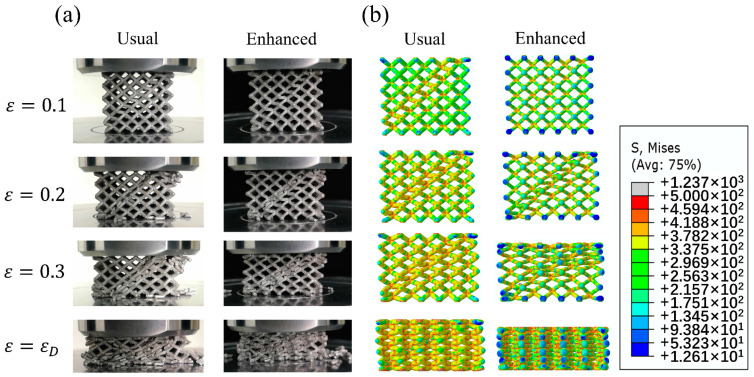
Deformation process of usual lattice structure D1 and EP sample D4 (**a**) and corresponded Mises stress distribution (**b**).

**Figure 10:**
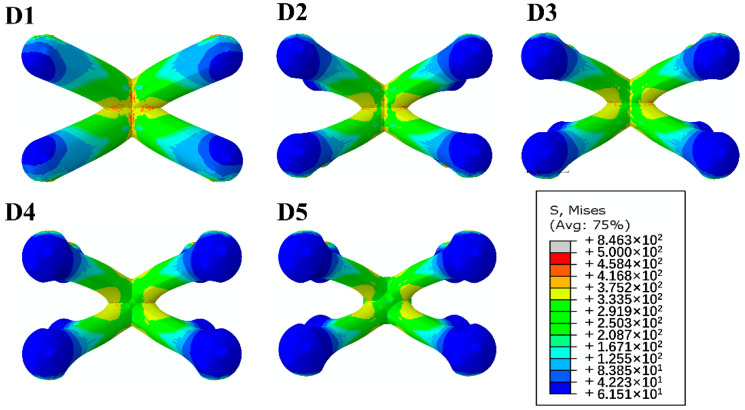
Mises stress distribution diagrams of a unit cell with different end diameters.

Figure 9a shows the compression deformation process of usual and enhanced lattice structures D1-D5, and Figure 9b is the corresponded Mises stress distribution diagrams. Since the failure modes of samples D1-D5 are similar, we use D1, i.e., a usual pyramidal structure and D4, i.e., the enhanced lattice structure with *d_e_* = 1.7 mm as the representatives to explain the compression process. It is seen that all the samples in Group D show multiple diagonal deformation bands during the compression. This suggests that the deformation of lattice structures arisen in a layer-by-layer mode, similar to that of dense metallic solids. The stress distribution diagram is shown in Figure 9b demonstrates that there does exist obvious stress concentration near the nodes. However, as shown in Figure 10, the enhanced structures D2, D3, D4 and D5 exhibit largely decreased stress concentration in comparison of the usual pyramidal lattice structure D1. Meanwhile, the enhanced structures also show continuously increased load-bearing ability of struts with increasing the *d_e_*.

**Figure 11:**
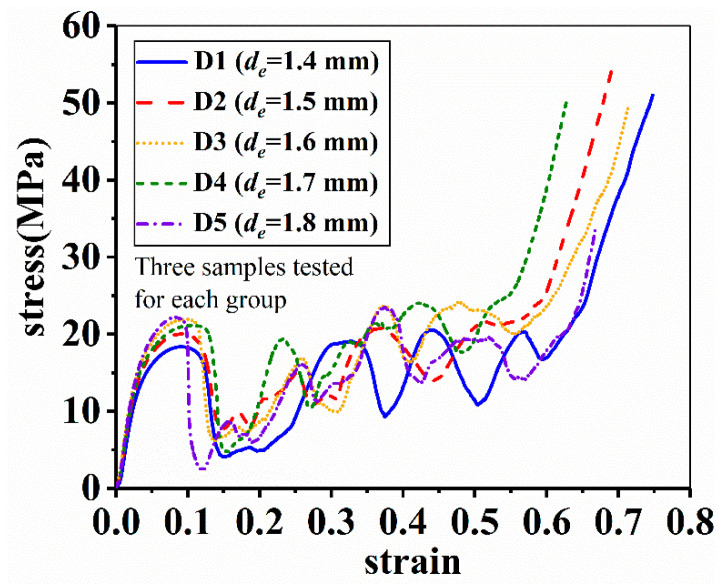
Effect of end diameter on the compressive stress–strain behavior of samples.

Figure 11 shows the compressive stress–strain curves of samples at varied *d_e_*, i.e., end diameters of struts. Like other porous materials, the lattice structures also exhibit three-stage stress–strain behavior, namely the elastic, plateau and densification stage. However, there is a sharp drop after the elastic stage in the stress strain curves of lattice structures, followed by a seriously fluctuated plateau stage. The two characteristics are greatly different from those of ductile porous materials. These fluctuations should be ascribed to the formation of deformation bands in the lattice structures that lead to a temporary drop in the load-bearing ability of lattice structures.

It is also seen from Figure 11 that the yield strength of lattice samples roughly increased with increasing the *d_e_* value except for D3. The strength of D3 seems to be higher than D4 although the latter has a larger *d_e_*. Moreover, when the compression entered the plateau stage, the relationship between the stress and the *d_e_* value seems to be irregular. These uncertainties could be resulted from the complex deformation behavior of lattice structures and should be studied later.

### 3.2. Energy Absorption

The representing mechanical properties drawn from experimental and simulated results are listed in Table 3, where $\sigma_{P}$ and $E^{*}$ are the compressive strength and equivalent modulus; P is the efficiency of energy absorption that is calculated by the following formula [32] and thus $P_{max}$ is the maximum efficiency of energy absorption; $W_{vmax}$ is absorbed energy per unit volume until the end of plateau stage of stress strain curves, here, the strain at the highest energy absorption efficiency is adopted as the end of the plateau stage.

(1) $$P=\frac{\int_{0}^{\varepsilon }\sigma d\varepsilon }{\sigma_{P}}$$

From the data in Table 2, it is also seen that the simulated results are acceptable. The maximum errors of $\sigma_{P}$, $E^{*}$, $W_{vmax}$ and $P_{max}$ are about 24.05%, 18.81%, 26.22% and 25.61%, respectively.

**Figure 12:**
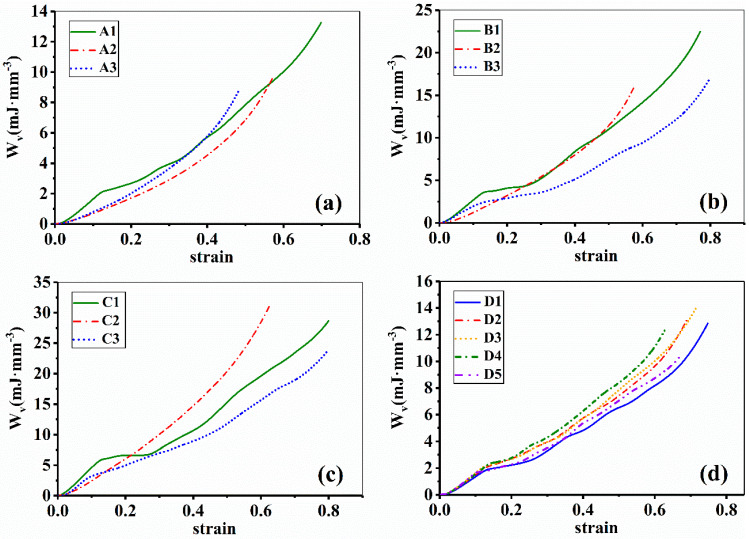
Absorbed energy per unit volume of EP lattice structure samples; (**a**–**d**) represents Group A–D, respectively.

Figure 12 shows the absorbed energy per unit volume *W_v_* against strain of all samples. It is clearly seen that different strut materials and different inclination angles lead to different energy absorption behaviors. When the strut material keeps unchanged but the inclination angle is increased, the energy absorption capacity will be greatly enhanced. For example, sample A1, B1 and C1 have the same strut material but different inclination angles. C1 shows the highest *W_v_* while A1 the lowest because they have the largest and the smallest inclination angle, separately.

On the other hand, if the strut material is the same, the *W_v_* increased with increasing the *d_e_* although there are some unexpected situations. For the sample D1 to D4, for example, the de is gradually increased from 1.40 mm to 1.70 mm, and correspondingly the *W_v_* is continuously increased from 8.72 mJ∙mm^−3^ to 10.13 mJ∙mm^−3^. Although D5 has the largest *d_e_*, it does not show the highest energy absorption capacity and its *W_v_* is only 9.35 mJ∙mm^−3^. This could be attributed to excessive difference between the de and dm, even if there is almost no stress concentration, the thin middle struts cause premature fracture, resulting in energy loss. This phenomenon indicates that there is a limit to the increase of energy absorption by node enhancement, and it should continue to be studied in the future.

As expected, the higher the strength of strut material, the higher the energy absorption capacity of lattice structures. As is seen from Figure 1 and Figure 12, the AM AlSi10Mg alloy has the highest strength and thus shows the strongest energy absorption capacity, and for the same reason, the as-cast AlSi10Mg alloy has the lowest strength and thus shows the weakest energy absorption capacity. A previous study published by Wang et al. [33] showed that the ordered porous aluminum cubic structures prepared by ZL111 alloy exhibits better energy absorption and bearing capacity than pure aluminum. This shows the influence of matrix material state on the mechanical properties, which is consistent with the results of this study.

**Figure 13:**
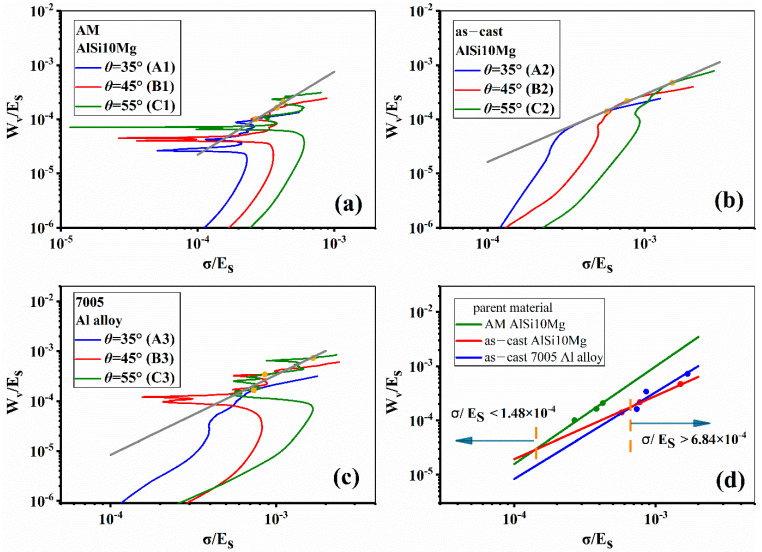
Energy absorption diagrams of samples with different strut materials and inclination angles; (**a**–**c**) represent the results of AM AlSi10Mg, as–cast AlSi10Mg and 7005 Al alloy, respectively; (**d**) is the summary of above results.

In order to compare the energy absorption behavior among the three strut materials, the energy absorption diagrams have been established based on the scale relationship between $W_{vmax}/E_{S}$ and $\sigma /E_{S}$ in a logarithmic coordinate system, as shown by Figure 13a–d, in which $E_{S}$ is the Young’s modulus of strut material. According to the trajectory rule proposed by Maiti et al. [34], there is an optimal energy absorption point or shoulder point in the energy absorption diagram for each sample. Enveloping the points in different inclination angles for each sample will form a straight line. The slope of straight line and its intercept with coordinate axes indicate the energy absorption capacity of lattice structure in a certain stress level. Figure 13a–c separately show the envelope lines for the three alloy lattice structures with varied inclination angles, and Figure 13d is the summary of three envelope lines. It is seen from Figure 13d that the sample made of as-cast AlSi10Mg alloy shows the best energy absorption performance when $\sigma /E_{S}<1.48\times 10^{-4}$. However, when stress level is over this value, the AM AlSi10Mg alloy lattice structure has the highest $W_{Vmax}/E_{S}$.

### 3.3. Prediction of Mechanical Parameters

#### 3.3.1. Calculation of $\sigma_{P}$ and $E^{*}$

Gibson and Ashby [1] proposed a relationship between the compressive strength and relative density of foams by a phenomenological method. This relationship has been proved to be applicable for lattice structures by researchers [35,36,37]. However, the related equations and studies have not involved the change of inclination angle of multi-layer lattice structures. To solve this problem, the following model is proposed by including the effect of inclination angle on the compressive strength of lattice structures.

Supposing that a lattice structure is subjected to a downward vertical pressure F, then the maximum plastic moment $M_{P}$ is proportional to $FL_{e}$, i.e.,

(2) $$M_{P}\propto FL_{e}cos\theta$$

For a circular cross-sectional strut, the moment is equal to

(3) $$M_{P}=\frac{d^{3}}{6}\sigma_{s}$$

where d is the diameter of strut such as $d_{e}$ in this study and $\sigma_{s}$ is the yield strength of strut material.

The F and strength σ meet the following relationship

(4) $$F\propto \sigma {\left(L_{e}cos\theta \right)}^{2}$$

Substituting Equations (3) and (4) into Equation (2), $\sigma_{P}$ can be obtained:

(5) $$\frac{\sigma_{P}}{\sigma_{S}}\propto \frac{d_{e}{}^{3}}{L_{e}{}^{3}}\cdot \frac{1}{cos^{3}\theta }$$

In the elastic deformation stage of lattice structures, the strain ε and deflection δ follow the following relationships, respectively:

(6) $$\varepsilon =\frac{\delta }{L_{e}sin\theta }$$

(7) $$\delta \propto \frac{FL_{e}{}^{3}}{E_{S}I}$$

#### 3.3.2. Calculation of $W_{vmax}$

As is known, when plastic deformation occurs, plastic hinges will be formed near the nodes of struts in lattice structures, and as a result, the applied energy is transformed to the rotation energy of struts. For pyramidal lattice structures, the rotation angle of plastic hinge during the deformation is just the inclination angle θ [38].

Considering a unit cell, the energy absorbed by all plastic hinges, $W_{1}$, can be expressed as:

(8) $$W_{1}\propto 2\theta M_{P}$$

The apparent volume of a unit cell, $V^{*}$, can be calculated by:

(9) $$V^{*}=2L_{e}{}^{3}sin2\theta cos\theta$$

Combining Equations (8) and (9), $W_{vmax}$ can be produced:

(10) $$\frac{W_{vmax}}{\sigma_{S}}\propto \frac{d_{e}{}^{3}}{L_{e}{}^{3}}\cdot \frac{\theta }{sin2\theta cos\theta }$$

#### 3.3.3. Validation of Theoretical Results

In this study, $L_{e}$ and $d_{e}$ are considered as constants because only the effect of θ is examined. In addition, due to the complexities of geometry and deformation behavior of lattice structures, the relationship between the mechanical properties of lattice structures and the inclination angle should be not simply proportional but more likely a linear function, therefore, two constants *K* and *R* are used to represent the slope and intercept, respectively. Equations (5), (8) and (10) can be separately modified as:

(11) $$\frac{\sigma_{P}}{\sigma_{S}}=K_{1}\frac{1}{cos^{3}\theta }+R_{1}$$

(12) $$\frac{E^{*}}{E_{S}}=K_{2}\frac{tan\theta }{cos\theta }+R_{2}$$

(13) $$\frac{W_{vmax}}{\sigma_{S}}=K_{3}\frac{\theta }{sin2\theta cos\theta }+R_{3}$$

where $K_{1}$, $K_{2}$, $K_{3}$ and $R_{1}$, $R_{2}$, $R_{3}$ are all geometrically dependent constants.

**Figure 14:**
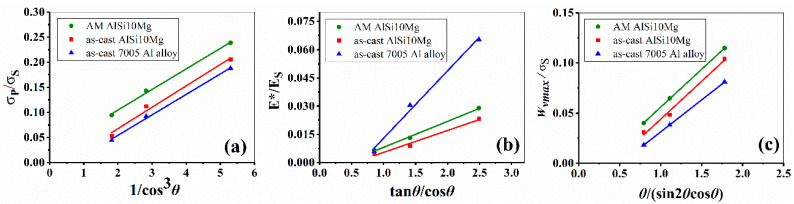
Theoretical results of mechanical properties against inclination angle; (**a**–**c**) are the theoretical relationship of angle with respect to compressive strength, equivalent modulus and energy absorption, respectively.

Substitute the relevant data in Figure 1 and Table 3 into Equations (11)–(13), Figure 14 gives the experimental and calculated results, and related data are listed in Table 3. It is clearly seen that all the fitted trajectories of mechanical parameters are straight lines, being consistent with the theoretical predictions. The constants K and R in Table 4 show certain discrepancies when the strut material changes. From the above equations, the mechanical parameters for a specific inclination angle of pyramidal lattice structures can be predicted.

## 4. Conclusions

The enhanced lattice structures in three strut materials are fabricated by two preparing methods. The influence of preparing methods, strut materials and geometric parameters on compression behavior and energy absorption is systematically investigated through finite element analysis and compression experiments. The theoretical expressions of relationship between three parameters and inclination angle are proposed. The main conclusions are summarized as follows:

(1) Compared with metallic additive manufacturing, the manufacturing method of 3D printing combined with investment casting eliminates stress fluctuations in plateau stages on stress–strain curves of pyramidal lattice structures and even increases energy absorption at large inclination angles. However, the compressive strength decreases at the same time.

(2) Increasing the inclination angle of enhanced pyramidal lattice structures narrows the gap in load-bearing capacity and improves energy absorption. For as-cast 7005 Al alloy lattice structures, brittle fracture occurs during the compression process, which causes energy loss.

(3) The end diameter of enhanced pyramidal lattice structures is crucial for compressive strength and energy absorption. Thickening the end diameter reduces the stress concentration near the node to a certain extent.

(4) The envelope line of shoulder points in energy absorption diagrams with respect to the inclination angle is a straight line. The energy absorption diagrams demonstrate that for AlSi10Mg, 3D printing combined with investment casting is a better choice when $\sigma /E_{S}<1.48\times 10^{-4}$, on the contrary, metallic additive manufacturing can be considered. The usage of as-cast 7005 Al alloy is a compromise solution when $\sigma /E_{S}>6.84\times 10^{-4}$.

(5) The theoretical equations and finite element analysis are consistent with the experimental results, which can be used to predict the mechanical and energy absorption properties of node-enhanced pyramidal lattice structures.

## Figures and Tables

**Table 1:** Parameters of lattice structures.

Group	*d_e_* (mm)	*d_m_* (mm)	Strut Material	*θ* (°)	*H* (mm)	$\rho^{*}$
A1	1.70	1.25	AlSi10Mg powder	35	5.00	0.3278
A2	1.70	1.25	as-cast AlSi10Mg	35	5.00	0.3278
A3	1.70	1.25	as-cast 7005	35	5.00	0.3278
B1	1.70	1.25	AlSi10Mg powder	45	6.16	0.3486
B2	1.70	1.25	as-cast AlSi10Mg	45	6.16	0.3486
B3	1.70	1.25	as-cast 7005	45	6.16	0.3486
C1	1.70	1.25	AlSi10Mg powder	55	7.14	0.3938
C2	1.70	1.25	as-cast AlSi10Mg	55	7.14	0.3938
C3	1.70	1.25	as-cast 7005	55	7.14	0.3938
D1	1.40	1.40	AlSi10Mg powder	35	5.00	0.3307
D2	1.50	1.34	AlSi10Mg powder	35	5.00	0.3415
D3	1.60	1.31	AlSi10Mg powder	35	5.00	0.3484
D4	1.70	1.25	AlSi10Mg powder	35	5.00	0.3278
D5	1.80	1.18	AlSi10Mg powder	35	5.00	0.3410

**Table 2:** Plastic parameters of three strut materials.

AlSi10Mg Powder		As-Cast AlSi10Mg		7005 Aluminum Alloy	
Plastic Strain	Stress (MPa)	Plastic Strain	Stress (MPa)	Plastic Strain	Stress (MPa)
0	116.94	0	113.91	0	119.03
0.0037	218.19	0.0126	149.27	0.0106	236.02
0.0077	255.68	0.0262	177.05	0.0210	262.08
0.0116	283.78	0.0396	187.70	0.0316	277.13
0.0156	307.31	0.0529	201.83	0.0421	289.14
0.0195	325.19	0.0661	211.70	0.0527	298.12
0.0234	338.82	0.0769	220.06	0.0632	304.61
0.0268	347.14	0.0919	227.16	0.0738	309.03
0.0334	357.75	0.1194	234.41	0.0843	311.99
0.0398	362.30	0.1345	243.31	0.0998	313.59

**Table 3:** Representing mechanical properties of samples.

Sample	$\sigma_{P}(\mathrm{MPa})$ Exp./Sim.	$E^{*}(\mathrm{MPa})$ Exp./Sim.	$W_{Vmax}$$\left(\mathrm{mJ}\cdot {\mathrm{mm}}^{-3}\right)$ Exp./Sim.	$P_{max}$(%) Exp./Sim.
A1	21.93/19.43	601.46/591.11	10.13/8.54	42.45/43.95
A2	9.55/10.89	185.49/197.56	5.60/5.07	24.46/28.21
A3	11.02/13.67	169.27/192.00	4.58/5.27	22.26/23.79
B1	33.05/31.94	1205.86/1432.18	15.04/14.42	42.98/45.16
B2	20.18/18.03	346.52/426.81	8.69/10.99	28.18/37.41
B3	23.07/22.99	931.93/940.00	9.59/8.79	40.23/38.23
C1	55.38/54.11	2662.91/2842.14	26.64/30.38	47.03/37.44
C2	37.00/34.53	925.26/1066.32	18.77/16.70	31.49/34.12
C3	47.12/44.33	1836.72/1670.97	20.38/23.35	43.24/51.75
D1	18.37/15.77	406.09/451.22	8.72/7.12	41.78/45.15
D2	20.06/16.27	535.66/604.17	8.81/6.98	40.23/42.90
D3	22.16/19.05	550.17/567.75	9.43/8.77	37.95/46.04
D4	21.93/22.43	601.46/591.11	10.13/8.54	42.45/43.95
D5	22.20/24.09	591.26/701.64	9.35/8.74	40.97/36.28

**Table 4:** Mathematical expressions of fitted trajectories.

Strut Material	$\sigma_{P}$	$E^{*}$	$W_{vmax}$
AM AlSi10Mg	$\frac{\sigma_{P}}{\sigma_{S}}=0.041\frac{1}{cos^{3}\theta }+0.023$	$\frac{E^{*}}{E_{S}}=0.014\frac{tan\theta }{cos\theta }-0.006$	$\frac{W_{vmax}}{\sigma_{S}}=0.075\frac{\theta }{sin2\theta cos\theta }-0.019$
as-cast AlSi10Mg	$\frac{\sigma_{P}}{\sigma_{S}}=0.041\frac{1}{cos^{3}\theta }-0.028$	$\frac{E^{*}}{E_{S}}=0.012\frac{tan\theta }{cos\theta }-0.006$	$\frac{W_{vmax}}{\sigma_{S}}=0.076\frac{\theta }{sin2\theta cos\theta }-0.032$
as-cast 7005 Al alloy	$\frac{\sigma_{P}}{\sigma_{S}}=0.043\frac{1}{cos^{3}\theta }\pm 0.018$	$\frac{E^{*}}{E_{S}}=0.036\frac{tan\theta }{cos\theta }+0.023$	$\frac{W_{vmax}}{\sigma_{S}}=0.064\frac{\theta }{sin2\theta cos\theta }-0.033$

## Data Availability

The data that support the findings of this study are available from the corresponding authors upon reasonable request.
